# Notes and News

**Published:** 1891-07-04

**Authors:** 


### Notes and News.

Collected and Communicated.

The Metropolitan Hospital Sunday Fund amounted to £39,000 on Wednesday, and there is every reason to believe that the total sum raised will equal if it does not surpass that of 1890. Although one congregation has been much advertised as having given the largest amount in the history of the Fund there are grounds for hope that Christ Church, Lancaster Gate, will beat the record this year.

Something like twenty per cent. more patients have been treated at the Abingdon Cottage Hospital during the year 1890 than were admitted in 1889. This in a great measure accounts for the balance in hand on December 31st, 1890, being only £62 2s. 8d. (on an expenditure of £422) as compared with one of £219 10s. 3d. at the end of 1889. Altogether 125 patients were admitted (29 of them accidents), and five deaths had occurred.

The annual meeting of the Ipswich Hospital brought to light some unsatisfactory details as to the sanitary state of part of the building; it is also to be regretted that the question of rooms for nurses still remains unsettled. If Ipswich is not careful it will find its hospital far behind the times and falling into disrepute. The expenditure has exceeded the income by £400, though the housekeeping is fairly economical, and the cost for repairs small. The hospital has lost a good friend in Sir Richard Wallace, and it is to be hoped that public interest will be more widely roused in aid of the institution, and that some life and go will be instilled into its methods.

The funds of the City Orthopædic Hospital benefited to the extent of £750 by the thirty-ninth anniversary dinner, held at the Holborn Restaurant, on the 24th ult., under the presidency of the Earl of Aberdeen. Over 61,000 cases of deformity of the feet have come to this hospital for treatment since its opening in 1851, of whom 2,255 were fresh cases admitted in 1890. An endeavour has been made for some time past to acquire the building in Hatton Garden in which the hospital is situated with the result that in March last the first part (£3,000) of the sum required (£9,000) was paid down, the remaining £6,000 being left on mortgage. The Committee now appeal to the public to aid them in their endeavours to enlarge and render permanent a charity which has conferred so much benefit on the deformed poor. A variation in the usual order of procedure at these dinners was made in the presentation by the Committee of the hospital to the trustees of a portrait of the surgeon (Mr. E. J. Chance).

The annual garden party given by the Committee of the Brompton Cancer Hospital took place on the 26th ult., in the grounds of the Hospital.

The Provident Surgical Appliance Society, established in 1872 for the purpose of supplying cripples in all parts of the United Kingdom with artificial limbs and surgical instruments, held their anniversary dinner at the Hotel Metropole, on the 25th ult., Mr. Vicary Gibbs in the chair. Six thousand one hundred and thirty-four appliances, or 634 more than had ever been distributed in any one year, represented the work of the society during 1890. Seven hundred pounds was added to the funds of the society as the result of this dinner.

His Royal Highness the Duke of Cambridge, K.G., who since 1850 has held the office of president of the institution, on the 26th inst. opened the new buildings at the London Hospital, the foundation stone of which he laid last year. That there were faults in the structural arrangements of the hospital has for some time been apparent to the management, who, as soon as they saw their way to it, set to work to remove them, and by the erection of the new buildings it is believed this has been accomplished. Briefly, the chief errors in the old building consisted in the fact that the operating theatre was totally inadequate for the requirements of so large a hospital; that no room existed for the purpose of clinical teaching; that the access to the hospital for severe accidents was difficult; that no proper accommodation was provided for the students; and that the chapel was wrongly placed, seeing that it blocked up the corridors on the first and second floors, prevented a proper circulation of air, and made it necessary to use the wards as passages of communication from one side of the building to the other. All these defects the new buildings remedy, as they provide a suitable approach and covered entrance, a receiving room, a clinical theatre, a new operating theatre, a large day room for the students, and a new chapel. Besides this, the authorities not being altogether satisfied with the sanitary condition of the hospital, enlisted the services of Dr. Louis Parkes, and on his report have made extensive improvements at a cost of £9,500. Thus, with the £16,000 spent on the new buildings, £25,500 has altogether been expended on these improvements.

There was a large number of visitors at the ceremony, the fair sex contributing no small quota to the gathering. H.R.H., who arrived at three o'clock, was received in the front entrance hall by the Chairman (Mr. E. Murray Ind), the Vice-Presidents, and the members of the House Committee. Thence he was conducted to the new receiving-room, where the remainder of the short ceremony of inauguration took place. In acknowledging the address presented by the Chairman from the House Committee, and in declaring the new building open, the Duke of Cambridge expressed his gratification at the great improvements which had been carried out. It only astonished him to think that so much had been done in the difficulties under which the management had been placed. It was easy, said his Royal Highness amidst cheers, to find fault with a great work, but to condemn it because a certain number of individuals thought that some portions of it might have been better done, seemed to him to be not only ungenerous, but unjust.

After an inspection of the buildings, the new chapel was dedicated by His Grace the Archbishop of Canterbury, who delivered an excellent address on the chastening influence of pain and suffering, during the course of which he strongly commended the splendid work accomplished by the London Hospital.

